# The Effect of Cr Addition on the Strength and High Temperature Oxidation Resistance of Y_2_O_3_ Dispersion Strengthened Mo Composites

**DOI:** 10.3390/ma17112550

**Published:** 2024-05-25

**Authors:** Haochen Guan, Chongshan Lv, Qingming Ding, Guangda Wang, Ning Xiong, Zhangjian Zhou

**Affiliations:** 1 School of Materials Science and Engineering, University of Science and Technology Beijing, Beijing 100083, China; 2 Advanced Technology & Materials Co., Ltd., Beijing 100081, China; 3 State Key Laboratory of Nuclear Power Safety Technology and Equipment, University of Science and Technology Beijing, Beijing 100083, China

**Keywords:** molybdenum, oxidation resistance, oxide dispersion strengthen, mechanical alloying

## Abstract

Y_2_O_3_ dispersion-strengthened Molybdenum (Mo) composites were prepared by the mechanical alloying of Mo and Y powders then consolidation by spark plasma sintering. The effects of Chromium (Cr) addition (0 wt. %, 5 wt. %, 10 wt. % and 15 wt. %, respectively) on the mechanical performance and high-temperature oxidation resistance of Mo-Y_2_O_3_ were investigated. The introduction of Cr had a significant influence on the mechanical property and oxidation resistance of the Mo-Y_2_O_3_ composite. The highest bending strength reached 932 MPa when the addition of Cr content was 5 wt. % (Mo–5Cr–1Y sample). This improvement is likely attributable to the dual mechanism of grain refinement and solid solution strengthening. Moreover, the Mo–5Cr–1Y sample showed the thinnest oxide layer thickness after high-temperature oxidation tests, and exhibited the best oxidation resistance performance compared with the other samples. First principle calculation reveals that Cr could improve the Mo–MoO_3_ interface bonding to prevent rapid spalling of the oxide layer. Meanwhile, Cr also facilitates the formation of the dense Cr_2_(MoO_4_)_3_ layer on the surface, which can inhibit further oxidation.

## 1. Introduction

Molybdenum is considered as an important candidate material for high-temperature environment applications, such as in small module nuclear reactors for space exploration, due to its attractive combination of physical properties including high melting point and good high temperature strength, low coefficient of thermal expansion and good thermal conductivity, low yield of sputtering and erosion, and high resistance to irradiation induced swelling [1,2,3,4]. The development of the aerospace industry also requires advanced high-temperature materials with higher performance. However, the further application of Mo was limited by its insufficient strength at elevated temperatures, brittleness characteristic, as well as poor oxidation resistance. To better meet the severe service conditions, there has been an interest in developing Mo alloys with excellent strength and ductility at both room and high temperature [5,6]. In the pursuit of enhancing the mechanical properties of molybdenum alloys, two primary obstacles are encountered, namely, brittle characteristics and poor oxidation resistance.

Mo alloys usually display a propensity for brittle cleavage fracture at room temperature due to the inherent brittleness of the Body-Centered Cubic (BCC) crystal structure. This phenomenon can be attributed to the dominance of screw dislocations in the plastic deformation of BCC metals [7,8]. The complex, non-planar core structure of these screw dislocations, combined with their elevated thermal activation energy, results in a reduced migration rate in BCC metals [9]. This reduction can precipitate stress concentration, which ultimately manifests as brittleness at room temperature. On the other hand, the embrittlement of Mo alloy is greatly influenced by the preparation process, especially caused by impurity elements at the grain boundaries (GBs). Interstitial elements, such as oxygen (O) and nitrogen (N), tend to accumulate at GBs [10,11,12,13]. This accumulation can weaken the GBs, leading to brittle fracture during deformation. Reducing the content of such impurities at GBs would be effective in improving the strength and ductility. For instance, Miller et al. suggested that the addition of carbon (C) and boron (B) could inhibit the segregation of O at GBs in molybdenum alloy; therefore, the tensile elongation was increased by 17%, approximately [14,15].

The strength of Mo alloys can be enhanced by introducing fine second-phase particles, which can pin the grain boundary and inhibit grain growth, leading to grain refinement [16,17,18]. The fine grains form a large number of grain boundaries, which effectively impede dislocation motion and stress concentration. Moreover, impurities may be transformed into a second phase through appropriate alloying design; therefore, the concentration of impurity elements at the grain boundaries can be significantly reduced, resulting in grain boundary strengthening [19]. The second-phase particles also hinder the movement of dislocations. Due to the high strength of the dispersed particles, dislocations tend to bypass them via the Orowan mechanism [20]. The dispersed particles apply a back stress on the dislocations, preventing them from moving and thus strengthening the material. The strengthening effect of the second phase depends on the size of particles, the inter-particle spacing, and the volume fraction of the dispersion particles.

Rare earth oxides are beneficial dopants, as they can refine the grains, and impede the dislocation movement. Numerous studies have demonstrated that the addition of rare earth oxides reduces the grain size of pure molybdenum from several hundred micrometers to several micrometers, achieving both strength and ductility improvement [21,22,23,24,25]. For example, Sun et al. reported that Mo alloys strengthened with nanoscale La_2_O_3_ exhibit yield strength over 800 MPa and tensile elongation as large as 40% at room temperature [26]. The particles, with an average diameter of approximately 80 nm, reduce the grain size of Mo alloys to approximately 0.5 μm. Moreover, Y_2_O_3_ is also a potential dispersant. Hu et al. prepared Mo alloys doped by Y_2_O_3_ with the ultra-fine grain size of 620 nm and high yield strength of 902 MPa [6].

Rare earth elements are known as strong oxygen-adsorbing elements. If using pure rare metals instead of rare earth oxides in Mo alloy, it can be speculated that the added rare earth element will absorb the impurity element O, which causes grain boundary embrittlement during the formation process; therefore, this effectively enhances the mechanical properties of Mo alloy. However, the related research and results are still relatively limited.

The other major challenges for the high-temperature application of Mo alloys concern their poor oxidation resistance, especially for oxide-dispersed strengthened molybdenum alloys (ODS-Mo) [27,28,29]. Yao et al. investigated the air oxidation behavior of ODS-Mo and pure Mo at 600 °C, and found that ODS-Mo exhibited higher weight gain than pure Mo, indicating reduced oxidation resistance [30]. This is attributed to the ultra-fine grain structure, which facilitates oxygen diffusion and increases the oxidation rate. Numerous studies have shown that chromium (Cr) could form a compact oxide film on the surface of stainless steel, which can prevent the further diffusion of O element in the alloy and enhance the oxidation resistance of stainless steel [31,32,33,34,35]. However, the effect of adding Cr on the performance of ODS-Mo alloys remains unclear and requires further exploration.

The goal of the current research is to investigate the effect of Cr addition on the strength and oxidation resistance of a Mo–Y composite. Mo–1Y composites with different Cr content were fabricated by mechanical alloying and spark plasma sintering. The microstructure was analyzed by advanced characterization techniques. Three-point bending strength was measured to compare the strength. High-temperature atmospheric oxidation at 600 °C was tested and compared. The first-principal calculation was performed to assist in explaining the antioxidant mechanism.

## 2. Experiments

Commercial Mo powder (with an average particle size ~2 µm and a purity of >99.8%), commercial Cr powder (with an average particle size ~2 µm and a purity of >99.8%) and nanoscale Y powders (with an average particle size ~50 nm and a purity of >99%) were used as the starting materials. Mo powder was provided by Chengdu Hongbo Molybdenum Industry Co., Ltd, Chengdu, China. The other powders were provided by Kerry New Materials (Beijing) Technology Co., Ltd, Beijing, China. The composition design of Mo alloys was shown in Table 1. A pure Mo material was also prepared for comparison.

**Figure 1:**
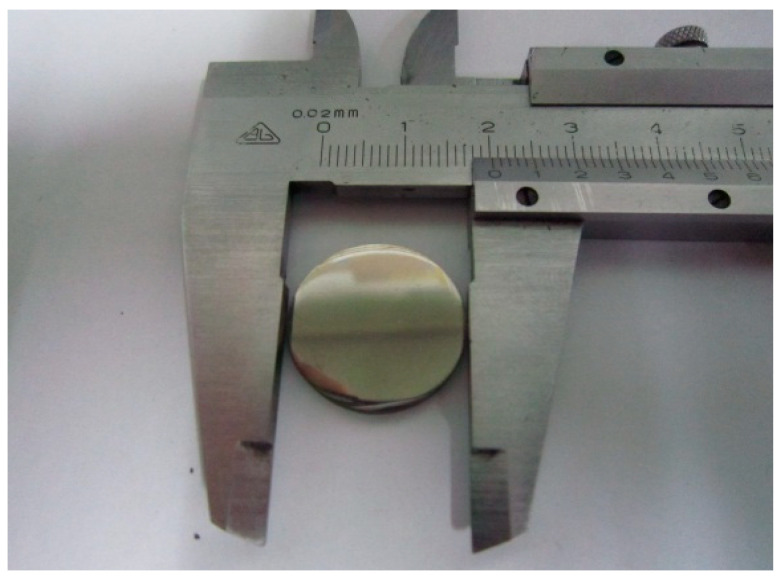
The polished sample after SPS.

The mixed powders were put into WC jars of 500 mL in volume, then mechanically alloyed (MA) in a horizontal planetary ball mill (QM-WX, Instrument Factory of Nanjing University, China) for 40 h under an argon atmosphere. The milling time and other milling parameters were determined via a literature review and an assessment of our previous works [17,36,37]. The material of the milling balls was TZM alloy. The diameters of the milling balls were 15 mm, 12 mm, 10 mm, 8 mm and 5 mm, with corresponding proportions of 10%, 10%, 25%, 25% and 30%, respectively. The weight ratio of milling balls to powders was 10:1 and the rotation speed was 400 rpm. Then, 20 g of the as-milled powders were pressed into a small cylindrical graphite mold (diameter, d = 20 mm), followed by spark plasma sintering (SPS) at 1500 °C and 50 MPa for 5 min. The heating rate of SPS was 100 °C/min. The sintered sample after polishing is shown in Figure 1.

The relative densities of the sintered samples were measured by the Archimedes method. The microstructures were investigated using scanning electron microscopy (SEM, Zeiss Gemini 300 Ultra, Berlin, Germany) with energy dispersive spectroscopy (EDS, Oxford Xplore 30 energy-dispersive, Oxford, UK). The second phase particles were characterized by transmission electron microscope (TEM, FEI, Tokyo, Japan). Selected area electron diffraction (SAED) was used to determine the phase of the dispersoid. The SEM samples were ground first using emery papers with grades of 240, 400, 600, 800, 1200 and 2000 grit size, respectively, then polished on polishing cloth using a diamond polish suspension of 3.5 μm as abrasive. Double spray thinning was used to prepare TEM samples. The voltage was 30 V, the temperature was −30 °C, and the electrolyte was a 10 vol.% HClO_4_ methanol electrolyte.

Three-point bending tests were performed on samples with an area of 15 mm × 3 mm and a thickness of 2 mm. The fracture surface morphology after the bending strength test was investigated by SEM.

Samples with a size of 4 mm × 6 mm × 8 mm were prepared for oxidation experiments. The samples were polished according to the SEM sample preparation process and their mass was measured (accurate to 0.01 mg) to calculate the weight gain after the oxidation test of the Mo alloys. The oxidation experiment was carried out in a muffle furnace without any protective gas. The heating rate was set to 8 °C/min, and then kept at 600 °C for 5 h. Finally, the furnace was cooled to room temperature, and the sample was weighed, to calculate the weight change of each sample after oxidation. The cross-section morphology of the oxide layer was observed by SEM and the composition of the oxide layer was measured by EDS. The D/MAX-2500 X-ray diffractometer (XRD, Rigaku, Tokyo, Japan) was used to analyze the phases of samples before and after oxidation tests. Each step lasted for one second and had a step width of 0.1°, with a scanning range of 0° to 100°.

Theoretical calculations involved in this work were performed using DS-PAW code [38] in the Device Studio program. Perdew, Burke, and Ernzerhof (PBE) was used for the exchange–correlation function of generalized gradient approximation with a plane wave cutoff of 500 eV [39]. All the supercells were optimized by structural relaxations, where the energy and force convergences were 10^−4^ eV and 10^−2^ eV/Å, respectively.

## 3. Results and Discussion

### 3.1. The Morphology and Phase of as-Milled Powders

**Figure 2:**
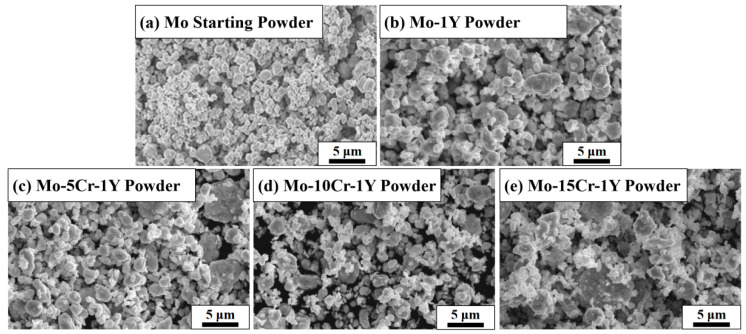
SEM of Mo powder before and after ball milling: (**a**) Mo starting powder; (**b**) Mo–1Y; (**c**) Mo–5Cr–1Y; (**d**) Mo–10Cr–1Y; (**e**) Mo–15Cr–1Y.

Figure 2 shows the morphology of the original Mo powders and the MA powders with different composition designs. The original Mo powder showed a spherical morphology with a narrow particle size distribution. After high-energy ball milling, the range of particle size distribution widened and the particle morphology became irregular. The particle size of fine powders was only around 100 nm, while the sizes of some large particles reached nearly 10 μm with a flat morphology. This phenomenon is more significant with the increase in Cr content. This is due to the phenomenon of “counter-grinding” during the MA process. In the process of high-energy ball milling, the powders will undergo severe plastic deformation under the collisions of ball and ball and ball and wall. On the one hand, the initial powders will be refined, while on the other hand, the crushed fine powders will undergo “micro forging” and repeated cold welding to form agglomerated particles with large sizes.

**Figure 3:**
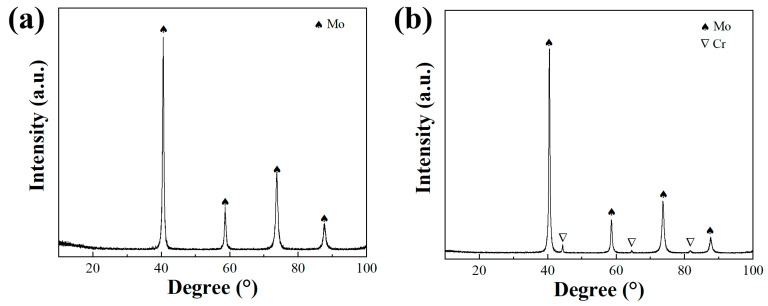
XRD of MA powders: (**a**) Mo–5Cr–1Y; (**b**) Mo–10Cr–1Y.

As shown in Figure 3, the XRD patterns of the as-milled Mo–5Cr–1Y and Mo–10Cr–1Y MA powders are presented. For the Mo–5Cr–1Y powder, the absence of distinct peaks corresponding to Cr and Y suggests an effective solid solution of both elements during the MA process. The minimal shift observed in the primary Mo peak is attributed to the relatively low Cr content, limiting its impact on peak displacement. In contrast, the Mo–10Cr–1Y powder exhibits pronounced Cr peaks, indicating the presence of elemental Cr within the powder. This occurrence is likely due to the addition of Cr exceeding the solid solubility of Mo during the MA process, resulting in an excess of Cr and the occurrence of Cr diffraction peaks.

### 3.2. The Microstructure and Mechanical Property after Sintering

**Figure 4:**
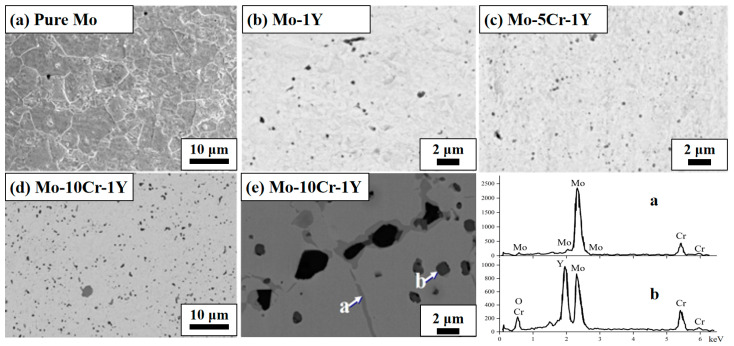
SEM of pure Mo and Mo–1Y with different Cr contents: (**a**) Pure Mo; (**b**) Mo–1Y; (**c**) Mo–5Cr–1Y; (**d**) low magnification SEM of Mo–10Cr–1Y; (**e**) high magnification SEM of Mo–10Cr–1Y.

Figure 4 shows the cross-section morphology of sintered materials with different composition designs. All samples show quite good sintering quality, and the absence of obvious pores or cracks. The relative densities of pure Mo, Mo-1Y, Mo–5Cr–1Y, Mo–10Cr–1Y and Mo–15Cr–1Y are 98.3%, 99.2%, 99.8% and 99.6%, respectively. The addition of Y and Cr are beneficial to densification during SPS. This agrees with our previous work on tungsten, showing that Y can facilitate the consolidation of refractory metal during SPS [36].

**Figure 5:**
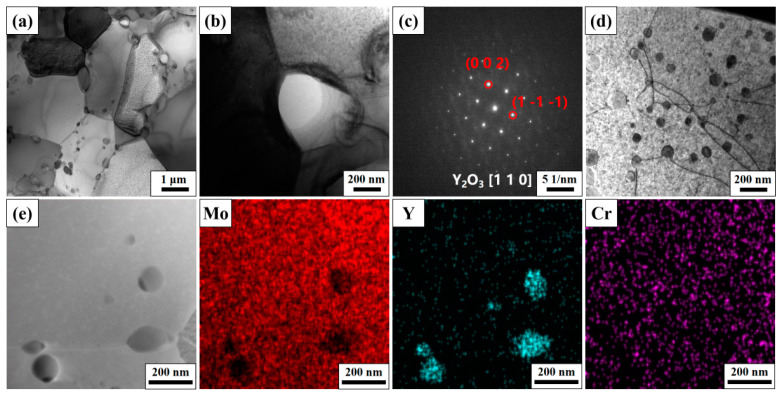
TEM of Mo–5Cr–1Y: (**a**) microstructure of Mo–5Cr–1Y; (**b**) an intergranular particle; (**c**) SAED of the intergranular particle; (**d**) intragranular particles interacting with dislocations; (**e**) EDS mapping scan results of the particles.

The pure Mo sample shows relatively large grain sizes, of several μm to around ten μm, as shown in Figure 4a, whereas the addition of Y and Cr refined the grain size of Mo obviously, to only hundreds of nm to several μm, as shown in Figure 5a. The grain refinement is probably due to the fine dispersal of second phase particles through grain boundary pinning mechanisms. As shown in Figure 4b,c,d, dispersed particles were found distributed in the matrix.

It should be noted that the size of dispersed particles increased obviously in the sample of Mo–10Cr–1Y, as shown in Figure 4d. SEM morphology at a higher magnification (Figure 4e) revealed the coexistence of two types of precipitates in Mo–10Cr–1Y alloy. One type appears as light-colored fine needle-shaped precipitates, as indicated by arrow a. The other type are dark-colored nearly spherical precipitates, as indicated by arrow b. EDS shows that the main component of needle-shaped precipitates is Cr-rich phases. Cr cannot be completely dissolved into the Mo matrix during the MA process when the addition of Cr increases to 10 wt. %, as indicated in Figure 3b. Therefore, the excessive amount of Cr will remain in the matrix as a Cr-rich phase. These light-colored Cr-rich phase also tends to become enriched around the dark-colored nearly spherical precipitates; according to EDS result, these spherical precipitates should be Y–Cr–O composites.

Figure 5 presents the TEM microstructure of the Mo–5Cr–1Y alloy. The grain of Mo–5Cr–1Y, as shown in Figure 5a, exhibits an average size of approximately 2 μm. Dispersed particles are observed both within grains and at grain boundaries. Notably, intergranular dispersed particles appear larger compared to those that are intragranular. In Figure 5b, the morphology of an intergranular dispersed particle at a triple grain boundary is illustrated, with a size of approximately 300 nm. Figure 5c shows the SAED result of the particle, which confirms that the particle is Y_2_O_3_. This result affirms our expectation that the addition of Y will consume the harmful impure oxygen. Our previous work on Y-alloyed tungsten also demonstrated this phenomenon [37]. Figure 5d shows the dispersed particle size within the grain. The interaction between these small intragranular dispersed particles and dislocations can be clearly seen, which impede the movement of dislocations, and indicate the typical bypass or cutting mechanism applied for strength improvement. The average size of these fine dispersed particles is around 50 nm. An EDS mapping analysis of the dispersed particles at the grain boundary and inside the grains is presented in Figure 5e. The dispersed particles are rich in Y, while there is no significant enrichment of Cr, indicating that Cr has been dissolved into the Mo matrix by MA and SPS. Therefore, this offers a benefit not only for sintering, but also for the improvement of mechanical properties.

**Figure 6:**
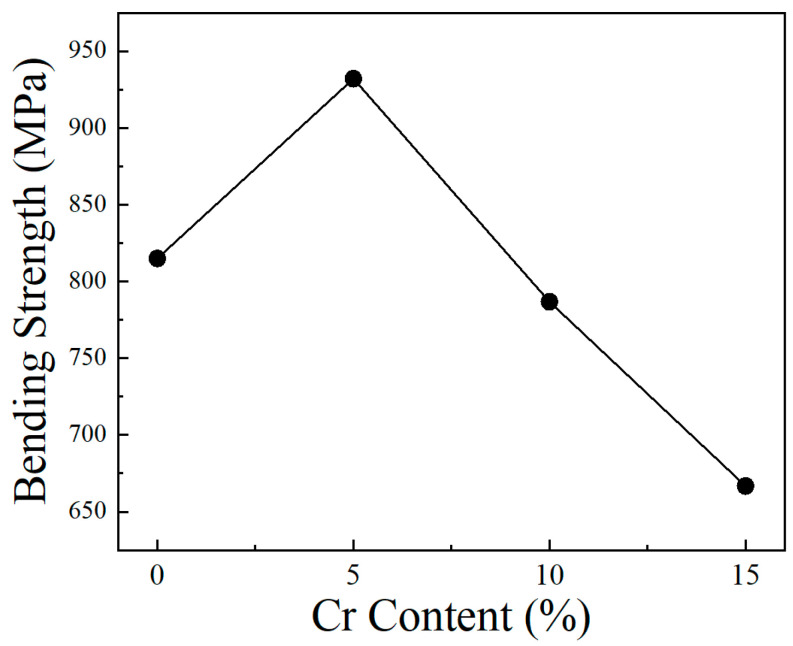
The effect of Cr content on the bending strength of Mo–xCr–1Y alloys.

Figure 6 shows the effect of Cr content on the bending strength of Mo–xCr–1Y alloys. Note that the bending strength of pure Mo is 549 MPa. After the addition of 1 wt. % Y, its bending strength increased obviously to higher than 800 MPa. These results demonstrate the strengthening effect of Y, as discussed above. Along with the Cr content increasing from 0 wt. % to 5 wt. %, the bending strength increased again from 815 MPa to 932 MPa. This enhancement can be attributed to the slight increase in relative density and the grain refinement caused by Cr elements in Mo alloys. Furthermore, Cr contributes to solid solution strengthening. However, when the addition of Cr content increases to 10 wt. %, the bending strength decreases to 786 MPa. When the Cr content is 15 wt. %, the bending strength further decreases to 667 MPa. The bending strength decrease along with the further increase in Cr content is likely attributable to the formation of large Cr-rich phases, which exhibit weaker interfaces within the Mo matrix, facilitating stress concentration and crack initiation.

**Figure 7:**
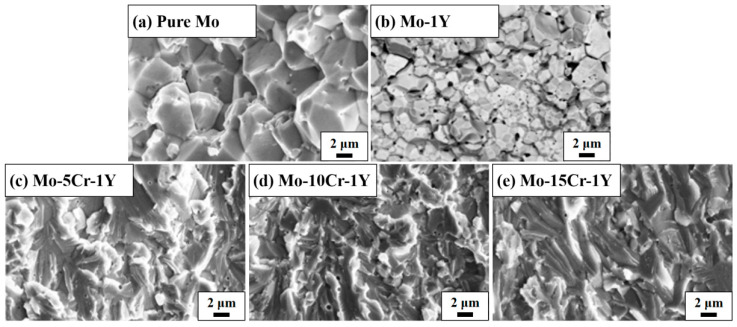
Fracture morphology of Mo–xCr–1Y alloys: (**a**) pure Mo; (**b**) Mo–1Y; (**c**) Mo–5Cr–1Y; (**d**) Mo–10Cr–1Y; (**e**) Mo–15Cr–1Y.

Figure 7 shows the fracture morphology after a bending test. An obvious transition from a typical brittle fracture morphology to quasi-cleavage fracture morphology, and then to a typical brittle river-pattern morphology, can be clearly seen. Pure Mo shows typical intergranular fractures, as shown in Figure 7a. The grain size is around 10 μm. For the Mo–1Y sample, the grain size is much finer than that of pure Mo. It shows a mixed fracture mode; both intergranular and trans-granular fractures can be found in Figure 7b, which is due to the pinning effect of dispersed particles. For the Mo–5Cr–1Y sample, a mixed brittle and toughness fracture feature can be found, as shown in Figure 7c, while with the further increase in Cr content, a typical brittle river-pattern fracture morphology can be found (Figure 7d,e).

### 3.3. High Temperature Oxidation Behavior

**Figure 8:**
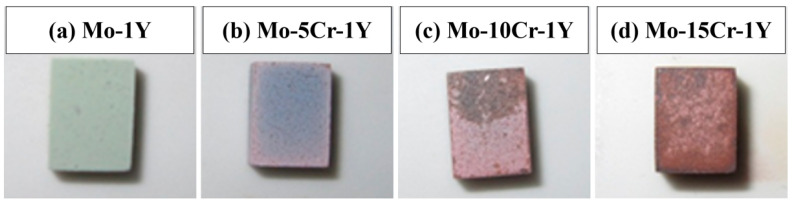
High-temperature oxidation morphology of Mo–xCr–1Y: (**a**) Mo–1Y; (**b**) Mo–5Cr–1Y; (**c**) Mo–10Cr–1Y; (**d**) Mo–15Cr–1Y.

Figure 8 illustrates the surface morphology of Mo–xCr–1Y (x = 0, x = 5, x = 10, x = 15) alloys after exposure to a high temperature atmosphere at 600 °C for 5 h. The sample of Mo–1Y shows a light green surface, which is same color as that of pure Mo after oxidation at 600 °C. The surface color changes from light green to blue-gray after the addition of 5 wt. % Cr. Notably, there is no observable peeling of the oxide layer. With the further increase in Cr content, the surface color becomes reddish-brown, and the color becomes inhomogeneous.

**Figure 9:**
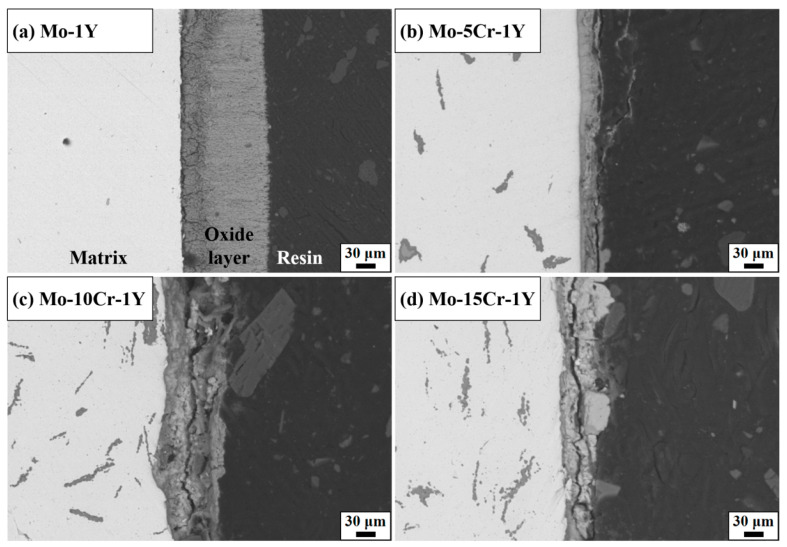
Cross-sectional oxide layer SEM of Mo–xCr–1Y: (**a**) Mo–1Y; (**b**) Mo–5Cr–1Y; (**c**) Mo–10Cr–1Y; (**d**) Mo–15Cr–1Y.

**Figure 10:**
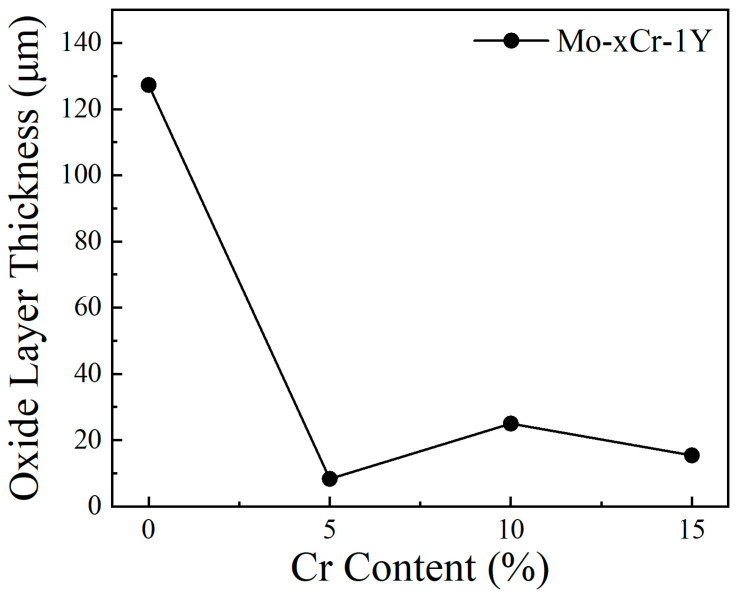
The relationship between oxide layer thickness and Cr content.

Figure 9 and Figure 10 depict the cross-section morphology and thickness of the oxide layer in samples with varying Cr contents. Mo–1Y displayed a thick oxide scale with a thickness of 127.3 μm. The interior of the oxide layer exhibited porosity and a lot of cracks. It seems that the oxide scale included two layers, a thin inner layer with a slightly black contrast and a thick outer layer. The darker inner layer may be composed of MoO_2_ and the lighter outer layer potentially comprises MoO_3_. It is well known that Mo is easily t oxidized in air when the temperature is higher than 400 °C, and the formed molybdenum oxide is porous and volatile, which cannot protect the matrix.

The thickness of the oxide scale obviously decreased after the addition of 5 wt. % Cr, indicating a significant improvement in oxidation resistance. The thickness of the oxide layer is only 8.3 μm. The oxide layer looks denser than that of the Mo–1Y sample. The interface between the substrate and the oxide layer appeared smoother, potentially attributable to Cr enhancing the interface bonding strength between the Mo matrix and the oxide layer. However, with the addition of 10 wt. % Cr, the interface between the matrix and the oxide scale became irregular. The thickness of the oxide layer increased to 25 μm. Obvious cracks can be seen in the oxide scale, which is likely due to a mismatch in elastic modulus between the matrix and the oxide layer. For the Mo–15Cr–1Y sample, although the thickness of the oxide scale seems thinner than that of the Mo–10Cr–1Y sample, the crack in the oxide layer is more dominant and penetrates along the entire oxide layer, which indicates the potential spalling of the oxide scale in samples with a high Cr content.

Based on the above results, it is clear that a suitable Cr content can improve the oxidation resistance of Mo alloy significantly. Cr also likely enhances the interface between the Mo matrix and oxide layer. To understand the strengthening effect of Cr on the Mo–MoO_3_ interface, the separation effect of the interface between Mo and MoO_3_ was calculated to reflect the fracture strength of the interface, which is defined as the energy needed for the separation of the phase boundary into two free surfaces, and can be calculated by the following formula [40]:

*W_sep_* = (*E_FS*1*_* + *E_FS*2*_* − *E_interface_*)/*S* (1)

where *E_FS_*_1_ and *E_FS_*_2_ are total energies of the two free surfaces, which are newly created after the fracture of the interface. The value of *W_sep_* could also be used to assess the impact of alloying elements on the ideal fracture strength of the interface.

**Figure 11:**
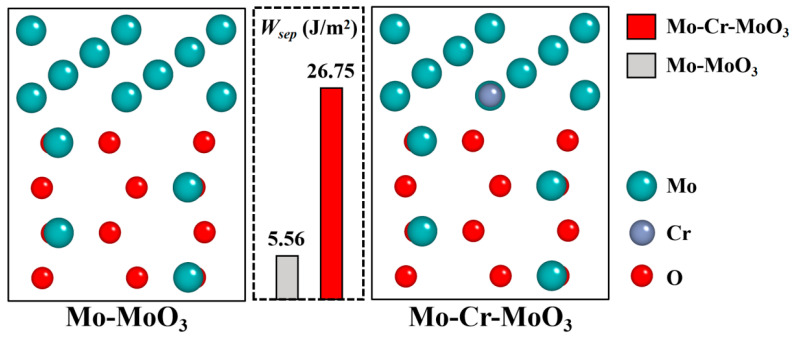
The interface model of Mo–MoO_3_ and Mo–Cr–MoO_3_.

The Mo–MoO_3_ and Mo–Cr–MoO_3_ interface model is shown in Figure 11. Compared with the Mo–MoO_3_ interface with a separation work of 5.56 J/m^2^, the separation work of Mo–Cr–MoO_3_ improved to 26.75 J/m^2^. The significant increase in separation work indicates that the presence of Cr significantly enhances the bonding strength between Mo and the oxide layer. This improved bonding is likely attributed to the stronger interaction between Cr and both Mo and MoO_3_. The strengthening effect of Cr is further supported by the observation that the oxide layer appears smoother with the addition of Cr, indicating a more uniform and dense oxide layer formation.

**Figure 12:**
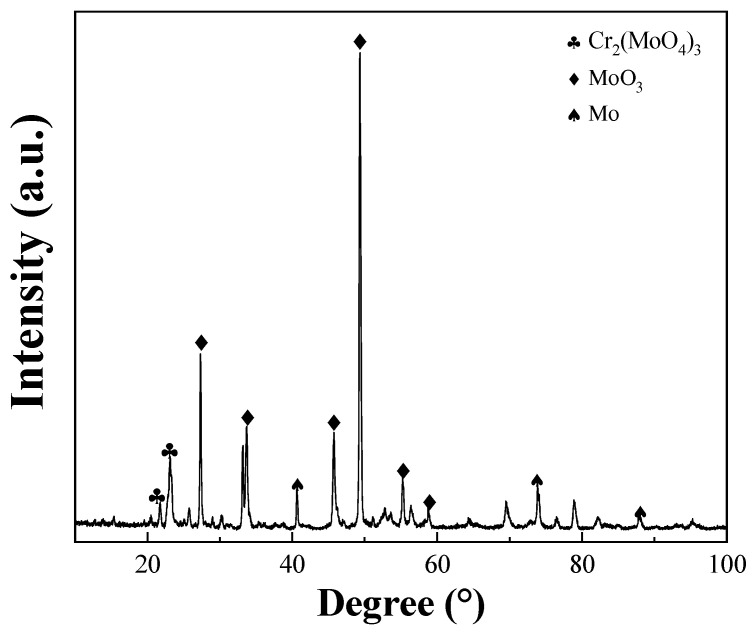
The oxide layer XRD of Mo–5Cr–1Y.

XRD was conducted on the oxide layer of the Mo–5Cr–1Y specimens, as shown in Figure 12. The oxide layer was found to predominantly consist of Cr_2_(MoO_4_)_3_ and MoO_3_. Previous research has indicated that at 600 °C, the Mo oxide layer primarily comprises MoO_3_, which volatilizes above 500 °C and melts at 795 °C [27]. At high temperatures, the anti-oxidation effect provided by the MoO_3_ oxide film is limited due to the vigorous volatilization of MoO_3_, resulting in significant weight loss of the substrate due to the severe oxidation of the Mo surface. Upon the addition of a certain amount of Cr, Cr_2_O_3_ will be formed and will react with MoO_3_ to produce a high melting point and dense protective layer of Cr_2_(MoO_4_)_3_ [41]. This protective layer effectively inhibits further oxidation of the Mo substrate, as evidenced by the oxidation reactions depicted in Equations (2)–(5).

Mo (s) + O_2_ (g) → MoO_2_ (s) (2)

2MoO_2_ (s) + O_2_ (g) → 2MoO_3_ (s) (3)

2Cr (s) + 3O_2_ (g) → 2Cr_2_O_3_ (s) (4)

Cr_2_O_3_ (s) + 3MoO_3_ (s) → Cr_2_(MoO_4_)_3_ (s) (5)

During the oxidation process, Mo reacts with O to form MoO_3_, while Cr reacts with O to produce Cr_2_O_3_ at the same time. Although the formation reaction of Cr_2_O_3_ has a lower Gibbs free energy and thus takes precedence, the limited amount of added Cr cannot completely prevent the formation of MoO_3_. Ultimately, at elevated temperatures, the reaction between Cr_2_O_3_ and MoO_3_ yields Cr_2_(MoO_4_)_3_, resulting in the formation of a high melting point and dense protective film. This film effectively inhibits the volatilization of MoO_3_ and the continued oxidation of the substrate.

In summary, the introduction of Cr into Mo–Y_2_O_3_ composites significantly enhances oxidation resistance compared to traditional Mo alloys, which often suffer from poor oxidation resistance at temperatures higher than 300 °C. The addition of Cr into Mo–Y_2_O_3_ composites resulted in a high bending strength of up to 932 MPa and the formation of a protective Cr_2_(MoO_4_)_3_ layer, which can inhibit the further oxidation of the matrix. A dense Cr_2_(MoO_4_)_3_ protective layer could not only prevent the rapid volatilization of MoO_3_, which is a common issue for traditional Mo alloys, but also inhibits the acceleration of oxygen diffusion and oxidation rate due to its fine grain microstructure, as also reported by Yao et al. [30].

## 4. Conclusions

This study investigated the effect of the addition of Cr on the mechanical properties and high-temperature oxidation resistance of Y_2_O_3_ dispersion-strengthened Mo alloys fabricated by MA and SPS. The main results can be summarized as follows:

(1) The addition of 1 wt. % Y can accelerate the densification of Mo and precipitate Y_2_O_3_ dispersion particles, thus clearly refining the grain size and strengthening the material. On this basis, adding a certain amount of Cr will further enhance the mechanical property. The Mo–1Y material with the addition of 5 wt. % Cr gave the highest bending strength of 932 MPa. However, when the addition of Cr content was higher than 10%, the bending strength was decreased, likely due to the formation of Cr-rich phases that weaken the Mo matrix, promoting crack initiation and growth;
(2) High-temperature oxidation experiments at 600 °C demonstrated that Cr addition markedly improved the oxidation resistance of the prepared Mo alloys compared with traditional Mo alloys. The optimal performance was observed in the Mo–5Cr–1Y composite, which exhibited a significantly reduced oxide layer thickness and smooth interface between the Mo matrix and the oxide layer, as compared to the Cr-free composite. Cr not only improved the Mo-MoO_3_ interface bonding, but also facilitated the formation of a dense Cr_2_(MoO_4_)_3_ layer on the surface, which can inhibit further oxidation.

## Figures and Tables

**Table 1:** Composition design of Mo alloys, wt. %.

Sample	Mo	Y	Cr
Mo	100	-	-
Mo-1Y	99	1	-
Mo–5Cr–1Y		1	5
Mo–10Cr–1Y		1	10
Mo–15Cr–1Y		1	15

## Data Availability

Data are contained within the article.
